# Synthesis and antimicrobial activity of β-lactams in dentistry for treatment of root canal infection

**DOI:** 10.1186/1471-2334-12-S1-P83

**Published:** 2012-05-04

**Authors:** M Gowri, R Rajesh, W Sofi, R Raghunathan, V Ganesh

**Affiliations:** 1 Department of Human Genetics, Sri Ramachandra University, Porur, Chennai-600 116, India; 2 Department of Organic Chemistry, University of Madras, Guindy Campus, Chennai-600 025, India

## Background

The treatment of root canal infection consists of eradicating microbes from the root canal and preventing re infection by root filling. Though the existing intra canal medicaments fight against multidrug resistant microbes in root canal, still re-infections occurs. The objective of this study is to synthesize new β-lactam compounds and evaluate their antimicrobial activity to treat root canal infections.

## Materials and methods

A series of β-lactam compounds were synthesized through 1, 3 dipolar cycloaddition reaction and their antimicrobial activity was evaluated against *E. faecalis*, *S. aureus*, *S. pneumoniae* and *C. albicans* that are commonly implicated in endodontic failures. The antibacterial activities were assessed *in vitro* by 1) Agar diffusion test (ADT) 2) MIC by microdilution method 3) Time kill assay 4) Efficacy in *ex vivo* dentine model 5) Haemolytic assay.

## Results

In this study, 16 compounds were tested and 6 compounds showed activity against *E. faecalis*, *S. aureus*, *S. pneumoniae* and *C. albicans.* In the time kill assay, the CFUs of *E. faecalis* were reduced after treatment with the compounds at their MICs. All the 6 compounds showed good antibacterial activity in dentinal tubule model at depth of 200 *μ*m and 400 *μ*m and the compounds were found to be hemocompatible.

## Conclusion

Overall, our experimental results revealed that β-lactam compounds exhibited promising antimicrobial activities in dentinal tubule model which can be further explored for the development of potent drugs against microbes involved in endodontic failures.

